# A rare recurrence of hydatid cyst with chest wall invasion: a case report

**DOI:** 10.3389/fmed.2026.1808294

**Published:** 2026-04-30

**Authors:** Hüseyin Çakmak

**Affiliations:** Department of Thoracic Surgery, Dr. Abdurrahman Yurtaslan Ankara Onkoloji Research and Training Hospital, Ankara, Türkiye

**Keywords:** dog tapeworm, *Echinococcus granulosus*, hydatid cyst, rib destruction, surgery, thoracic wall

## Abstract

**Background:**

Hydatid cyst is a zoonotic infection caused by the larval stage of *Echinococcus granulosus*. While the lungs are frequently affected, rib involvement and chest wall invasion are extremely rare, occurring in less than 2.5% of cases. We report a rare case of recurrent hydatid disease exhibiting infiltrative growth into the costal trabeculae and lung parenchyma.

**Case presentation:**

A 50-year-old man presented with weight loss. He had a history of splenectomy for hydatid disease 14 years prior. Physical examination and blood tests were unremarkable; however, chest computed tomography (CT) revealed a 56 × 52 mm multilocular cystic lesion in the apico-posterior segment of the left lung, causing destruction of the second, third, and fourth ribs. Immunoglobulin hemagglutination was positive (1/256). During left posterolateral thoracotomy, an 8 × 6 cm lesion was found prolapsing through the intercostal space. Due to the lack of a clear cleavage plane in the parenchyma and extensive rib destruction, an en bloc resection was performed, including a pulmonary wedge resection and partial resection of the three affected ribs. Histopathology confirmed the diagnosis of a hydatid cyst with daughter cysts and bone trabeculae involvement. The patient was discharged on the 10^th^ postoperative day without complications and started on albendazole (800 mg/day).

**Conclusion:**

This case demonstrates that hydatid cysts can recur many years after the initial surgery and may exhibit an atypical, infiltrative progression into neighboring tissues, including bone. While parenchyma-preserving surgery is the standard, radical en bloc resection may be necessary for curative treatment in cases of chest wall invasion. Long-term postoperative antihelmintic therapy is essential to prevent further recurrence.

## Introduction

Hydatid cyst, or cystic echinococcosis, is a severe zoonotic infection caused by the larval stage of the tapeworm *Echinococcus granulosus*. Humans act as accidental intermediate hosts, contracting the disease through fecal-oral ingestion of eggs from contaminated food, water, or soil, often linked to infected dogs and sheep (1). The lung is the second most commonly affected organ in adults, after the liver. Thoracic involvement typically presents with pulmonary cysts, while chest wall and rib involvement remains extremely rare (2). Bone hydatidosis accounts for 0.5–2.5% of cases, with rib involvement being particularly uncommon (2). This report describes a rare case of pulmonary hydatid disease with aggressive chest wall invasion and rib destruction. Although lung involvement is common, extension into the thoracic cage necessitates a radical surgical approach. We aim to highlight the importance of recognizing these infiltrative cases, where en bloc resection of the affected ribs is often required to achieve a complete cure.

## Case

**Figure 1:**
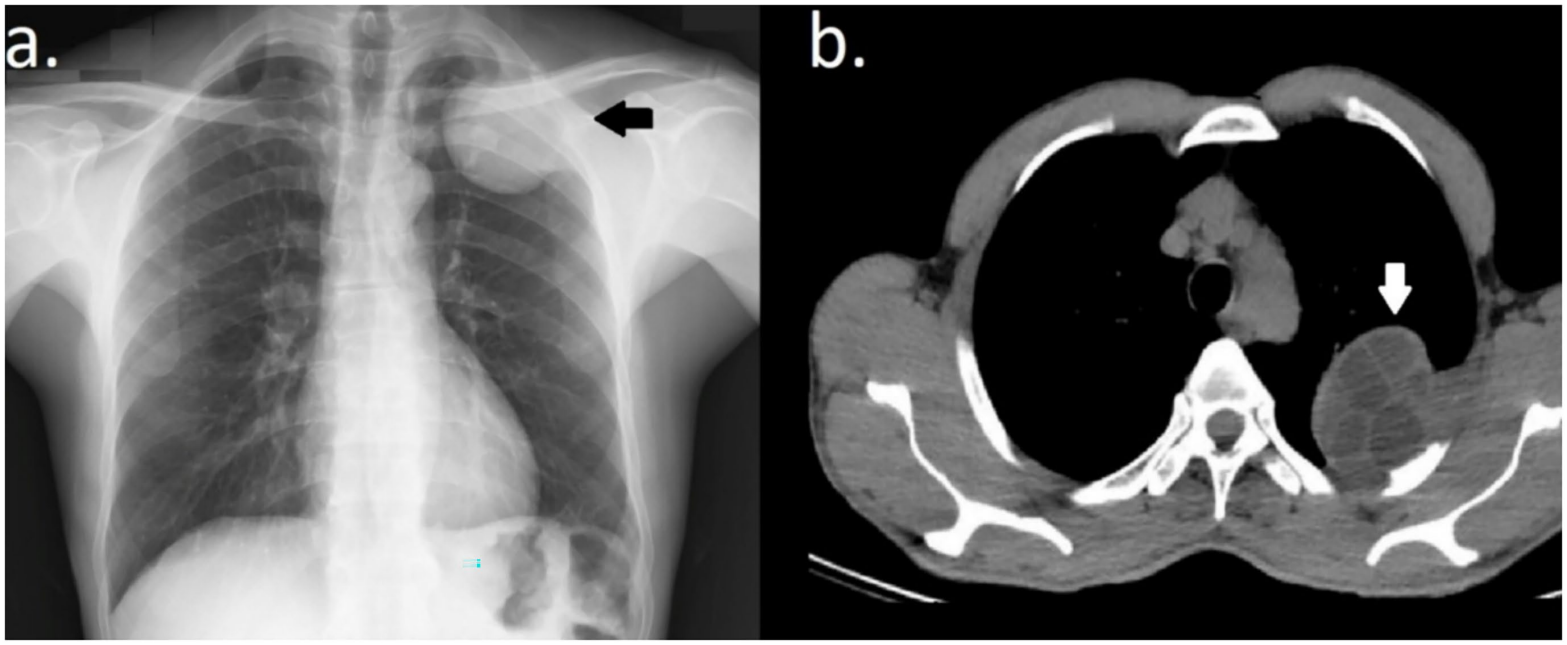
**(a)** Chest X-ray reveals a wide-based mass located on the costal pleura in the upper zone of the left lung (black arrow). **(b)** A multilocular cystic lesion with a thick wall, smooth contours, measuring 56 × 52 mm, and containing a few millimetric calcifications, was detected in the apico-posterior segment of the left lung on thorax CT, causing destruction on the second, third, and fourth costae (white arrow).

**Figure 2:**
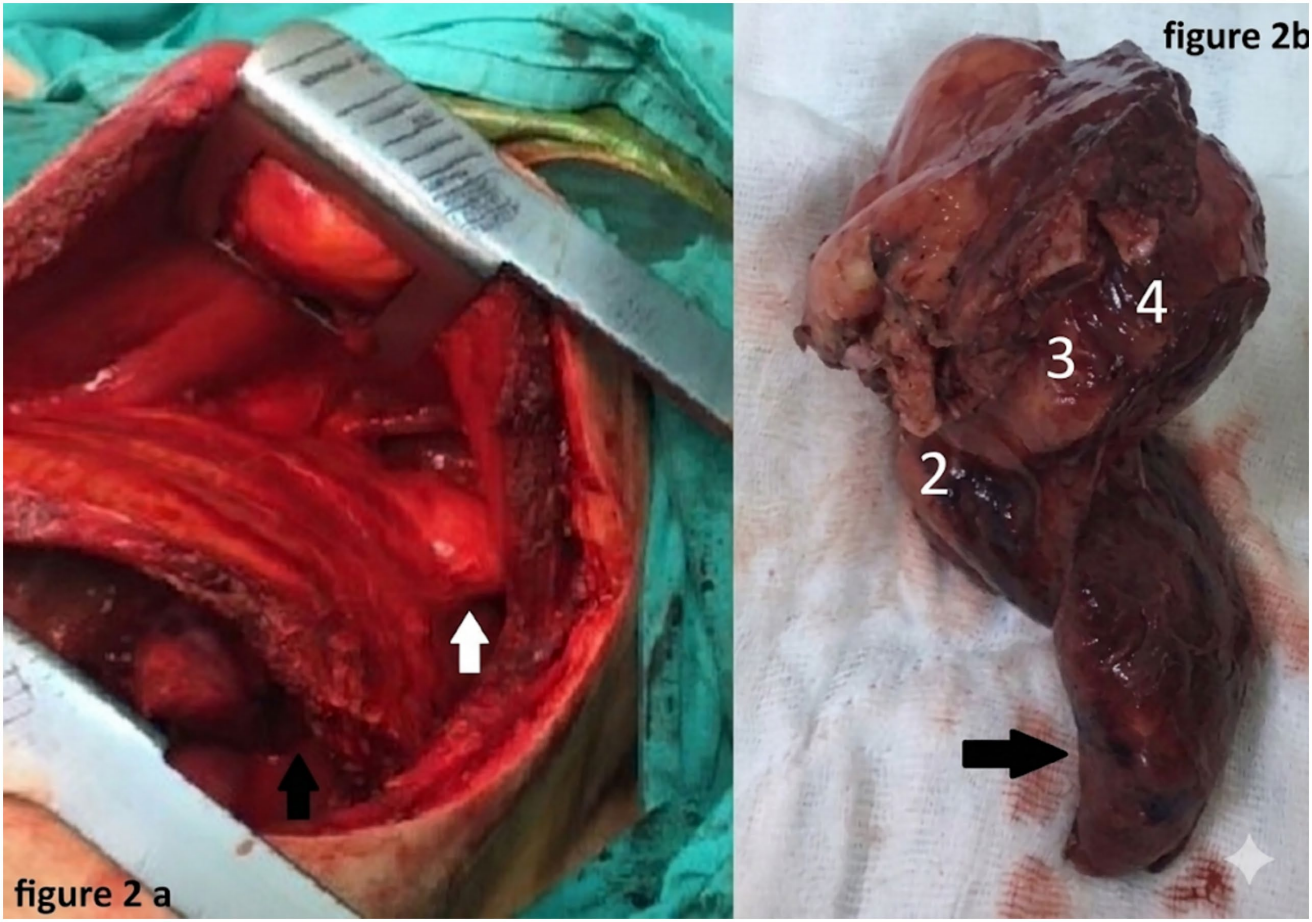
**(a)** Intraoperative view of the lesion with smooth contours, extended from the upper lobe of the left lung (black arrow) to the pleural space and protruded out of the thorax through the second intercostal space (white arrow). **(b)** Image of en-bloc resection material; the lung is marked with a black arrow, and the ribs are marked with white numbers.

The male patient, aged 50 years, presented to the outpatient clinic with a complaint of weight loss. The non-smoking patient worked for a shipping company and had a history of splenectomy for a hydatid cyst 14 years ago. The patient received albendazole therapy at a dose of 400 mg twice daily for three 21-day cycles. No pathological findings were detected in the patient in the physical examination and routine blood tests; however, in the chest X-ray, a lesion in the upper zone of the left lung adjacent to the thoracic wall was detected, as shown in Figure 1a. Therefore, the patient was referred to a chest surgery clinic for consultation. In thoracic CT, a multilocular cystic lesion with a thick wall was detected in the apico-posterior segment of the left lung with smooth contours, sized 56 × 52 mm, causing destruction of the second, third, and fourth costae, located centrally and peripherally, and containing a few millimetric calcifications as shown in Figure 1b. The lesion was evaluated as a type 3 hydatid cyst or cystic necrotic tumor. When the immunoglobulin hemagglutination test results of the patient were found to be 1/256 positive, with a diagnosis of a hydatid cyst, left posterolateral thoracotomy was performed. During exploration, a lesion with smooth contours sized 8 × 6 cm, extending from the left lung upper lobe to the pleural space and prolapsing out of the thorax through the second intercostal space, was detected as exposed in Figure 2a. When the parenchyma was dissected, it was consistent with hydatid cysts, but the cyst wall was not freed from the lung tissue. To evaluate the relationship between the cystic lesion and the apex and to prevent uncontrolled perforation, wedge resection was applied to a limited area in the upper lobe apico-posterior segment. In subsequent exploration, it was determined that the posterior parts of the costae were destroyed by the cyst. The lesion was excised en bloc with wedge resection applied to the thoracic wall (second, third, and fourth costae partial resections) and the left upper lobe of the lung (Figure 2b). As the defect in the thoracic wall was superposed to the scapula, no graft was placed.

**Figure 3:**
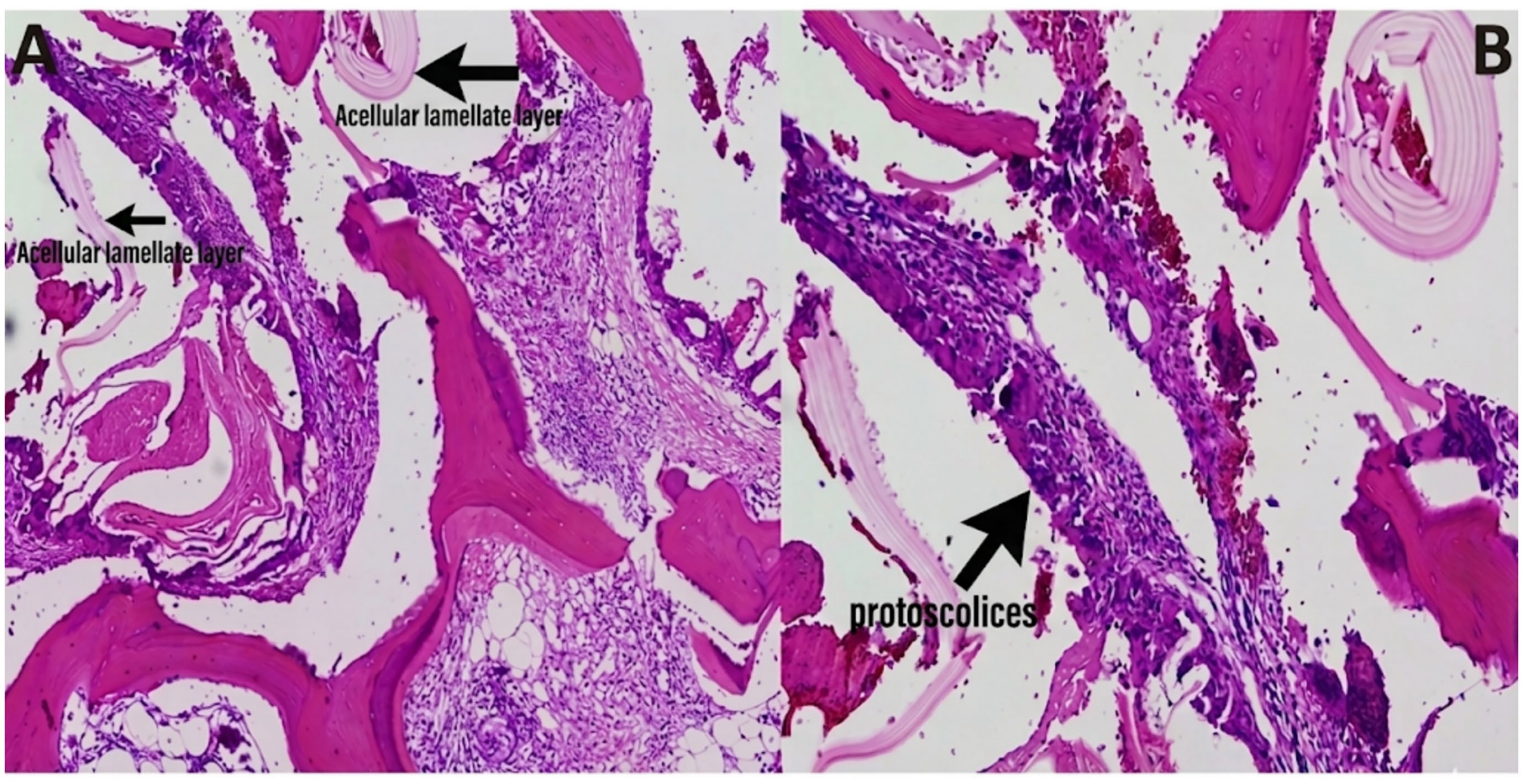
**(a)** Cystic lesion that contains cuticular/lamellar eosinophilic material between bone lamellae (indicated by arrows) (X100 hematoxylin eosin staining) and **(b)** hematoxylin eosin staining appearance of bone intra tissue cystic lesion and protoscolices (X200).

The histopathological examination revealed fragments of the proliferating membrane, daughter cysts, protoscolices, multinucleated giant cells, inflammatory infiltrates, and trabeculae of spongy bone tissue. Histological appearance confirmed the diagnosis of hydatid cyst disease (Figure 3).

The patient had normal respiratory and cardiovascular function during the postoperative period. Moderate pain in the area of the wound was present, and the wound healed without complications such as paradoxical breathing. At postoperative fifth and eighth days, chest tubes were removed. The patient was referred to the infectious diseases clinic and started on antiparasitic chemotherapy with albendazole tablets 400 mg × 2/day. The patient was discharged on the 10th postoperative day. The patient has been followed up for 25 months postoperatively without any evidence of recurrence. The clinical course and management timeline of the patient are summarized in Table 1.

**Table 1:** Clinical course and management timeline of the patient.

**Date**	**Event**	**Outcome**
2010	Initial diagnosis and splenectomy	Primary surgery for splenic hydatid cyst
2010–2024	Latency period	Asymptomatic follow-up (no recurrence noted)
February 2024	Onset of symptoms	Left-sided chest pain and dyspnea
February 2024	Clinical presentation	Computed tomography (CT) revealed the recurrent mass
March 2024	Surgical intervention	En bloc resection of ribs and lung-preserving surgery
Post-op day 10	Discharge	Patient discharged with albendazole treatment
Follow-up	25-month check-up	No signs of early local recurrence

## Discussion

Pulmonary hydatid cysts usually require a course of treatment that includes surgical intervention and removal of the infected areas. Surgery may vary depending on the size and location of the cyst and the patient’s overall health. The aim of this operation is to completely remove the cyst and prevent recurrence. However, appropriate anthelmintic therapy and other supportive measures are often necessary before and after surgical interventions. Therefore, the effective treatment of pulmonary hydatid cysts involves a comprehensive, multidisciplinary approach, including surgical intervention. Parenchyma-preserving surgeries, such as cystotomy and capitonnage, are generally accepted procedures. Although surgeons are advised to avoid radical procedures, radical surgical treatment may be required in patients presenting with atypical localization (3).

The thoracic wall is an uncommon location for hydatid cysts, even in endemic areas (4). Bone hydatid cysts are very rare, between 0.5 and 2.5%, and frequently involve the spine, long bones, and pelvis (5). Isolated rib involvement is extraordinary, with –an incidence estimated at 0.18–1.21% (6). Involvement of the ribs is extremely rare, with fewer than 100 cases reported in the literature (7). Rib hydatidosis can be primary or secondary due to spontaneous rupture of a pulmonary or mediastinal cyst or puncture of a subpleural location (6, 7). However, in our case, there has been no complaint related to a ruptured hydatid cyst since the diagnosis of the disease 14 years ago. In the exploration performed during the surgery, no pleural band, thickening, infection symptoms, or cystic lesions in the pleural space, suggesting secondary spread, were observed. This suggested that the disease may have spread from the lung parenchyma to the thoracic wall or from the thoracic wall to the lung parenchyma.

The disease, which forms pericystic tissue by suppressing peripheral tissue in the parenchyma, progresses infiltratively in the bone. This suggests that the source of the disease was the source that caused the infection that required splenectomy to be performed 14 years ago. However, the disease in the lung and costae, which went unnoticed in the examinations performed at that time, showed that the involvement was too limited to be detected.

The growth of the parasite in the bone is a very slow process. The osseous structure of the rib restricts parasitic growth, although there is no pericystic formation (7). However, cysts in the parenchyma grow rapidly owing to their soft tissue and form pericystic tissue by suppressing neighboring tissues.

The presence of a lesion in the adjacent parenchyma, the absence of pericystic tissue observed in normal hydatid cyst cases, and the hydatid cyst picture with a totally invasive appearance suggest that the disease might have an alternative mechanism of spread. It seems impossible to determine whether the disease originated from the parenchyma or the bone. Nevertheless, regardless of the tissue where the disease spreads, this suggests a possible pathway of spread through neighboring areas in the muscle and connective tissue that form the bone, lung parenchyma, and thoracic wall, causing destruction, which is not consistent with its normal progression. During the operation, we detected the destruction of three neighboring costae and diffuse microscopic cystic lesions in the neighboring parenchyma and intercostal space. This suggests that, unlike the known infection and spread of the disease, it can spread through neighboring tissues in line with the structure of the tissue in which it exists or the structure of the neighboring tissues and that it can display different behaviors in the tissues in which it is located.

## Conclusion

This case demonstrates that hydatid disease may recur after a prolonged latency and can exhibit aggressive, infiltrative behavior involving bone and adjacent structures. Although lung-preserving techniques are preferred, radical en bloc resection may be necessary in cases with chest wall invasion. Long-term postoperative antiparasitic therapy remains essential to reduce the recurrence risk.

## Data Availability

The original contributions presented in the study are included in the article/supplementary material, further inquiries can be directed to the corresponding author.
